# Corticosteroid inhibits differentiation of palmar fibromatosis-derived stem cells (FSCs) through downregulation of transforming growth factor-β1 (TGF-β1)

**DOI:** 10.1371/journal.pone.0198326

**Published:** 2018-06-26

**Authors:** Jung-Pan Wang, Hsiang-Hsuan Michael Yu, En-Rung Chiang, Jir-You Wang, Po- Hsin Chou, Shih-Chieh Hung

**Affiliations:** 1 Department of Surgery, School of Medicine, National Yang-Ming University, Taipei, Taiwan; 2 Department of Orthopedics & Traumatology, Taipei Veterans General Hospital, Taipei, Taiwan; 3 Department of Radiation Oncology, H. Lee Moffitt Cancer Center and Research Institute, Tampa, Florida, United States of America; 4 Integrative Stem Cell Center, China Medical University Hospital, Taichung, Taiwan; Boston University Henry M Goldman School of Dental Medicine, UNITED STATES

## Abstract

Treatment for musculoskeletal fibromatosis remains challenging. Surgical excision for fibromatosis is the standard therapy but recurrence remains high. Corticosteroids, an anti-fibrogenic compound, have been used to treat early stage palmar fibromatosis, but the mechanism is unknown. We investigated the inhibitory mechanism effect of corticosteroids in the murine model of fibromatosis nodule as well as in cultured FSCs. Quantitative reverse transcription/polymerase chain reaction (PCR) analysis and immunofluorescence (IF) staining for markers of myofibroblasts (α-smooth muscle actin and type III collagen) were used to examine the effect of dexamethasone on myofibroblasic differentiation of FSCs both in vitro and in vivo. Transforming growth factor-β1 (TGF-β1) signaling and its downstream targets were examined using western blot analysis. TGF-β1 expression in FSCs before and after dexamethasone treatment was compared. In addition, inhibition of TGF-β1 expression was examined using RNA interference (RNAi) on FSCs, both in vitro and in vivo. Treating FSCs with dexamethasone inhibited FSCs’ myofibroblastic differentiation in vitro. Treating FSCs with dexamethasone before or after implantation further inhibited formation of fibromatosis nodules. Dexamethasone suppressed expression of TGF-β1 and pSmad2/3 by FSCs in vitro. TGF-β1 knockdown FSCs showed reducing myofibroblastic differentiation both in vitro and in vivo. Finally, addition of TGF-β1 abolished dexamethasone-mediated inhibition of myofibroblastic differentiation. Dexamethasone inhibits the myofibroblastic differentiated potential of FSCs both in vitro and in vivo through inhibition of TGF-β1 expression in FSCs. TGF-β1 plays a key role in myofibroblastic differentiation.

## Introduction

Musculoskeletal fibromatosis is a benign soft tissue tumor with aggressive behavior. It can be divided into superficial (fascial) and deep (musculoaponeurotic) groups. Superficial fibromatosis typically presents as subcutaneous nodules formed by rapid myofibroblast proliferation followed by slow involution to dense acellular fibrosis. An example of this condition is palmar fibromatosis (Dupuytren’s disease) [1]. Recently we reported isolation of fibromatosis stem cells (FCSs) from palmar fibromatosis [2], and postulated a murine model of fibromatosis nodule by implanting isolated FSCs to the back skin of nude mice [3]. The FSCs differentiated into myofibroblasts two weeks after implantation. After two months following implantation, fewer myofibroblasts were noted and type I collagen was evident. This model recapitulates clinical course of fibromatosis: from the proliferative stage, then the involutional stage, to the residual stage.

TGF-β is a multifunctional growth factor involved in cell proliferation, differentiation, and extracellular matrix protein synthesis [4]. It also plays a significant role in various diseases associated with fibrotic changes, such as liver fibrosis [5]. When TGF-β1 binds to TGF-β receptor (TβR) II, transcription factors Smad2 and Smad3 phosphrylate and create a transcription complex that regulate transcription of specific genes including α-smooth muscle actin (α-SMA), collagen type III (Col III), and collagen type I (Col I) [4]. In our previous study, we demonstrated that TGF-β1 enhanced the fibrogenesis of FSCs in vitro and suggested that TGF-β1 plays a role in developing fibromatosis and its recurrence by increasing the expression of α-SMA and types III and I collagen [2]. Other investigators have also reported higher level of TGF-β1 in palmar fibromatosis [6] and penile fibromatosis [7] compared to normal tissues. However, the exact mechanism that mediates increase of endogenous TGF-β1 in fibromatosis is not clear and needs further investigation.

Surgical excision is the main treatment for late stage palmar fibromatosis [8]. Currently there is no consensus on non-operative treatment for early stage palmar fibromatosis. Gamma-interferon has been reported to reduce size of early stage palmar fibromatosis lesions [9]. Calcium channel blockers such as nifedipine or verapamil and prostaglandins E1 and E2 may decrease symptoms [10]. Corticosteroid has also been shown to inhibit early stage palmar fibromatosis [11, 12], but the exact mechanism of how corticosteroid inhibits fibromatosis is not clear. In this study, we investigated the inhibitory mechanism of dexamethasone on FSCs both in vitro and in vivo and the role of TGF-β1 in this mechanism.

## Materials and methods

### Isolation and expansion method

This research followed the tenets of the Declaration of Helsinki. The study protocol and written informed consent forms were approved by the Institutional Ethics Committee/Institutional Review Board of Taipei Veterans General Hospital, Taiwan. Following written informed consent, fibromatosis tissues were obtained from 3 patients with palmar fibromatosis. Isolation of FSCs was performed as previously described [3]. FSCs were grown in α-MEM (Invitrogen, Carlsbad, CA) which contains 10% fetal bovine serum (FBS; Invitrogen. Lot selected for rapid growth), 100 U/ml penicillin (Invitrogen), 100 μg/ml streptomycin (Invitrogen), and 250 ng/ml amphotericin B (Invitrogen). The medium was changed every 2 days and subcultured at 1:5 at subconfluence before cell growth reached 80% of confluence.

### Cell proliferation assay

To determine the inhibitory concentrations (IC50-values) of dexamethasone, cytotoxicity of dexamethasone was first measured with a cell proliferation test kit, 3-(4,5-dimethylthiazol-2-yl)-2,5- diphenyltetrazolium bromide (MTT, Sigma, St Louis, MO), to determine FSC cell death. The IC50-values were used as an indicator of proliferation inhibition. The cells were seeded at 2000 cells/well (in a 96-well culture plate) and incubated under different concentrations of dexamethasone (0, 002, 0.02. 0.2, 2, 20, 200 uM). Absorbance after incubation with MTT for 4 h at 37°C with 5% CO_2_ was measured by enzyme-linked immunosorbent assay (ELISA) plate reader after 7 days. Cell numbers were determined by the optical density (OD) value with a test wavelength of 560 nm.

### TGF-β1 knockdown by RNA interference (RNAi)

The expression plasmids and the bacteria clone for TGF-β1-shRNA (TRCN0000003317, TRCN0000003318) (S1 Table) were obtained from the National Science Council in Taiwan. pLKO. 1. TGF-β1-shRNA-puro is derived from pLKO.1-puro vector by inserting the shRNA oligonucleotide 5’-CCGG CCCG CGTG CTAA TGGT GGAA ACTC GAGT TTCC ACCA TTAG CACG CGGG TTTT T -3’ (TRCN0000003317) and 5’-CCGG CCGG CCTT TCCT GCTT CTCA TCTC GAGA TGAG AAGC AGGA AAGG CCGG TTTT T-3’ (TRCN0000003318) targeting human TGF-β1 sequence (CCCG CGTG CTAA TGGT GGAA A and CCGG CCTT TCCT GCTT CTCA T, respectively) into AgeI and EcoRI sites to generate a lentiviral expression vector. Cells transduced by lentivirus produced from this plasmid can be selected by puromycin. Lentivirus was produced by co-transfecting shRNA-expressing plasmids, envelope plasmids, and packaging plasmids into 293T cells using Lipofectamine 2000 (LF2000; Invitrogen, Carlsbad, CA, USA). Virus-containing supernatants were collected 48 h after transfection and were filtered. Subconfluent cells were infected with lentivirus in the presence of 8 ug/mL polybrene (Sigma-Aldrich, St Louis, MO, USA). At 24 h postinfection, the medium was removed and replaced with fresh growth medium containing puromycin (1 ug/mL) and selected for infected cells for 48 h. After puromycin selection and RNAi sequences testing for knockdown efficacy, the clone was used for in vitro and in vivo experiments(S1 Fig). All recombinant DNA research follows the National Institutes of Health guidelines.

### Western blot analysis

Cells were lysed in protein extraction reagent (M-PER, Pierce, Rockford, IL) plus protease inhibitor cocktail (Halt^™^; Pierce). Protein concentrations were determined using the bicinchoninic acid (BCA) assay (Pierce) and heated for 5 min at 95°C in a sample buffer. Equal aliquots of cell lysates were separated on a 10% SDS–polyacrylamide gel. Proteins were transferred to polyvinylidene fluoride (PVDF) membrane filters, blocked for 1 hour, and then probed with specific primary antibodies including pSmad (2/3) (Cell Signaling Technology, Beverly, MA), Smad7 (Santa Cruz Biotechnology, Europe), Sp1 (Santa Cruz Biotechnology, Europe), and β-actin (Novus Biologicals, Littleton, CO). The filter was washed and primary antibodies were allowed to bind by incubating it with horseradish peroxidase-conjugated goat anti-mouse or anti-rabbit IgG (BD Bioscience, San Jose, CA). The filter was washed again; proteins were detected using a chemiluminescence assay (Millipore, Billerica, MA).

### Reverse transcription (RT) and real-time polymerase chain reaction (PCR) analysis

Real-time RT-PCR was performed as described previously [3]. Briefly, random sequence primers were used to prime reverse transcription reactions and synthesis was carried out by Superscript III RT (Invitrogen, Carlsbad, CA). cDNA was synthesized from total RNA by means of M-MuLV reverse transcriptase. Real-time amplification of the genes was performed using cDNA as the template in a 20-μl reaction mixture containing LightCycler^™^–FastStart DNA Master SYBR green I (Roche Molecular Systems, Alameda, CA) and a specific primer pair of each cDNA on the ABI 7500 real-time PCR machine according to the manufacturer’s instructions (Applied Biosystems). To check the efficiency of PCR amplification and cDNA synthesis, human Glyceraldehyde-3-phosphate dehydrogenase (GAPDH) was used as an internal control (Table 1). Analysis was performed using the delta CT method included in software supplied by the machine.

**Table 1:** Primers used for real-time reverse transcription-polymerase chain reaction analysis.

Gene	Primer length	T_m_	Sense primer	Anti-sense primer
Col1A2	20	51.78	GACATGCTCAGCTTTGTGGA	CTTTCTCCACGTGGTCCTCT
Col3A1	20	50.2	GGAGAATGTTGTGCAGTTTG	AGGACCAGTAGGGCATGA
α-SMA	20	53.7	CATCATGCGTCTGGATCTGG	GGACAATCTCACGCTCAGCA
GAPDH	21	52.57	ATATTGTTGCCATCAATGACC	GATGGCATGGACTGTGGTCATG

Col1A2, α2 type I collagen; Col3A1, α1 type III collagen; α-SMA, α-smooth muscle actin; GAPDH, Glyceraldehyde-3-phosphate dehydrogenase

### In vivo murine model of fibromatosis nodule

The experimental protocols and animal care were in accordance with the institutional animal welfare guidelines of Laboratory Animal Center in Taipei Veterans General Hospital. All procedures involving animals were approved by the institutional animal care and use committee of Taipei Veterans General Hospital (IACUC 2012–082). Male athymic nu/nu mice were obtained (National Laboratory Animal Center, Taipei, Taiwan) at 4 to 6-week-old age. Mice were housed on arrival in a facility with a 12-h light/dark cycle. Food and water were provided ad libitum to mice and clean the waste 1–3 times a week. As described in our previous study, the murine model of fibromatosis nodules was created by implanting FSCs in 4 to 6 week-old male athymic nu/nu mice [3]. 1.0 × 10^6^ FSCs suspended in 200 μl of growth factor-reduced Matrigel (BD Biosciences, Bedford, MA) were injected subcutaneously into the back skin of a mouse. Each mouse received 4 injections. On Day 14, animals were sacrificed by CO_2_ Asphyxiation and Matrigel implants were harvested. The Matrigel implants were then fixed in 3.7% paraformaldehyde (PFA) and prepared for immunohistochemistry (IHC) and immunofluorescence (IF) staining.

### Hematoxylin & eosin staining and immunofluorescence staining

Paraffin-embedded sections were deparaffinized in xylene, dehydrated through graded alcohols from 100% to 70%, and then rinsed in ddH_2_O to remove organic solution. The sections were counterstained with Harris hematoxylin (Sigma-Aldrich, St. Louis, MO) and mounted. Sections of hematoxylin and eosin (H&E) staining were also used to analyze the Matrigel. The sections were observed by light microscope AX80 (Olympus) and image quantification was performed with ImageJ software.

For immunofluorescence, antigen retrieval was achieved by boiling the slides in 10 mM sodium citrate buffer pH 6.0 at 99°C for 30 min. The slides were then cooled to room temperature, rinsed in PBS, and blocked with 3% H_2_O_2_. Non-specific staining was blocked with PBS containing 5% heat FBS for 30 min at room temperature. Primary antibodies against α-SMA (Sigma-Aldrich, St. Louis, MO), type III collagen (Abcam, Cambridge, UK), and type I collagen (Abcam, Cambridge, UK) were placed on slides at appropriate dilutions at 4°C overnight, washed extensively with PBS, and then incubated with DyLight 594-conjugated goat anti-rabbit IgG (Jackson labs, West Grove, PA), DyLight 488-conjugated goat anti-rabbit IgG (Jackson labs, West Grove, PA), DyLight 594-conjugated goat anti-mouse IgG (Jackson labs, West Grove, PA) or DyLight 488-conjugated donkey anti-goat IgG (Jackson labs, West Grove, PA) secondary antibodies. The sections were counterstained with 4,6-diamidino-2-phenylindole (DAPI; Vector Labs, Burlingame, CA) for nuclear (blue) fluorescent staining. Immunofluorescence was observed with fluorescence microscope.

### Statistical analysis

Statistical analysis was performed using SAS statistical software (version 6.12, SAS Institute, Cary, NC) and SPSS software (version 8.0, SPSS, Chicago). Results are presented as means ± SD from at least three independent experiments. Statistical differences between experimental and control groups were determined by analysis of variance, and the criterion of significance was set as *p* < 0.05 by Student’s t-test.

## Results

### Dexamethasone inhibits differentiation of FSCs in vitro

**Fig 1:**
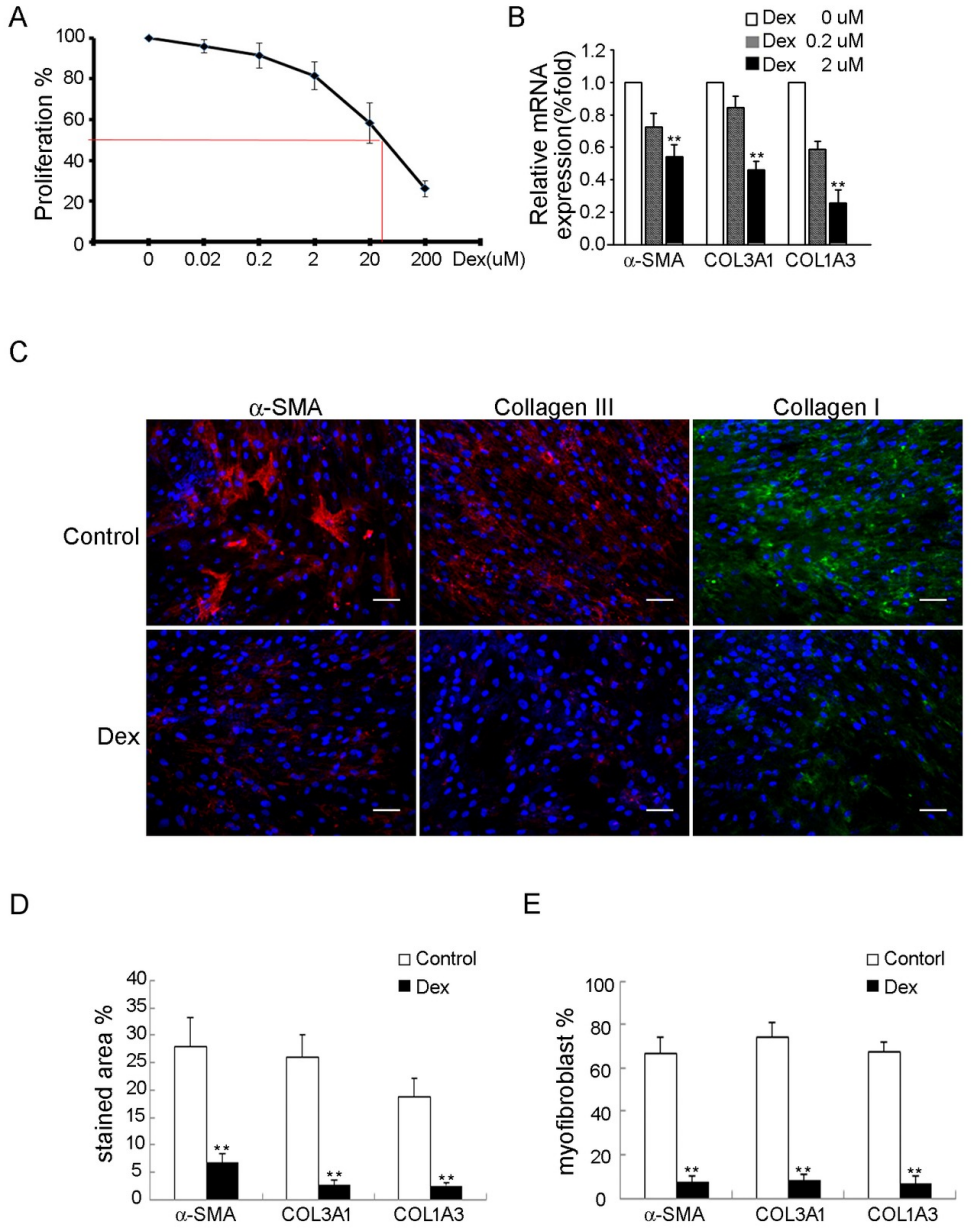
Dexamethasone inhibits in vitro myofibroblastic differentiation of FSCs. (A) FSCs seeded at 2000 cells/well in 96-well plates were treated with dexamethasone at the indicated concentrations for 7 days. The IC50-value was measured. (B) mRNA expression of α-SMA, Col3A1 and Col1A3 was analyzed by quantitative RT-PCR after treatment with dexamethasone 0, 0.2 and 2 uM for 14 days. (C) Immunofluorescence staining for α-SMA (red), types III (red) and I collagen (green) after treated with either dexamethasone 2uM or 0 uM (control) for 14 days. Bars = 50 μm. (D) The percentages of stained areas. (E) The percentages of myofibroblasts. Data are shown as mean ± SD (n = 3). Statistical significance is presented as **, *p*<0.01 compared with other groups. All experiments were repeated with FSCs isolated from three different donors.

Because dexamethasone is a known fibrogenic inhibitor, we assessed the effect of dexamethasone on fibrogenesis of FSCs. Dexamethasone did not induce significant cytotoxic effects at concentration up to 2 uM as assayed by MTT (Fig 1A). Interestingly, MTT assay revealed that dexamethasone at concentration up to 200 uM inhibited proliferation of FSCs in a dose dependent manner (Fig 1A).

Treatment with dexamethasone for 14 days inhibited the expression of α-SMA, Col3A1 and Col1A3 mRNAs in a dose dependent manner as demonstrated by quantitative RT-PCR (Fig 1B). Immunofluorescence also demonstrated that dexamethasone inhibited the expression of α-SMA and type III collagen (Fig 1C–1E). These data suggest that dexamethasone suppresses myofibroblastic differentiation of FSCs in vitro.

### Dexamethasone inhibits FSCs to form fibromatosis-like nodule in immunodeficient mice

**Fig 2:**
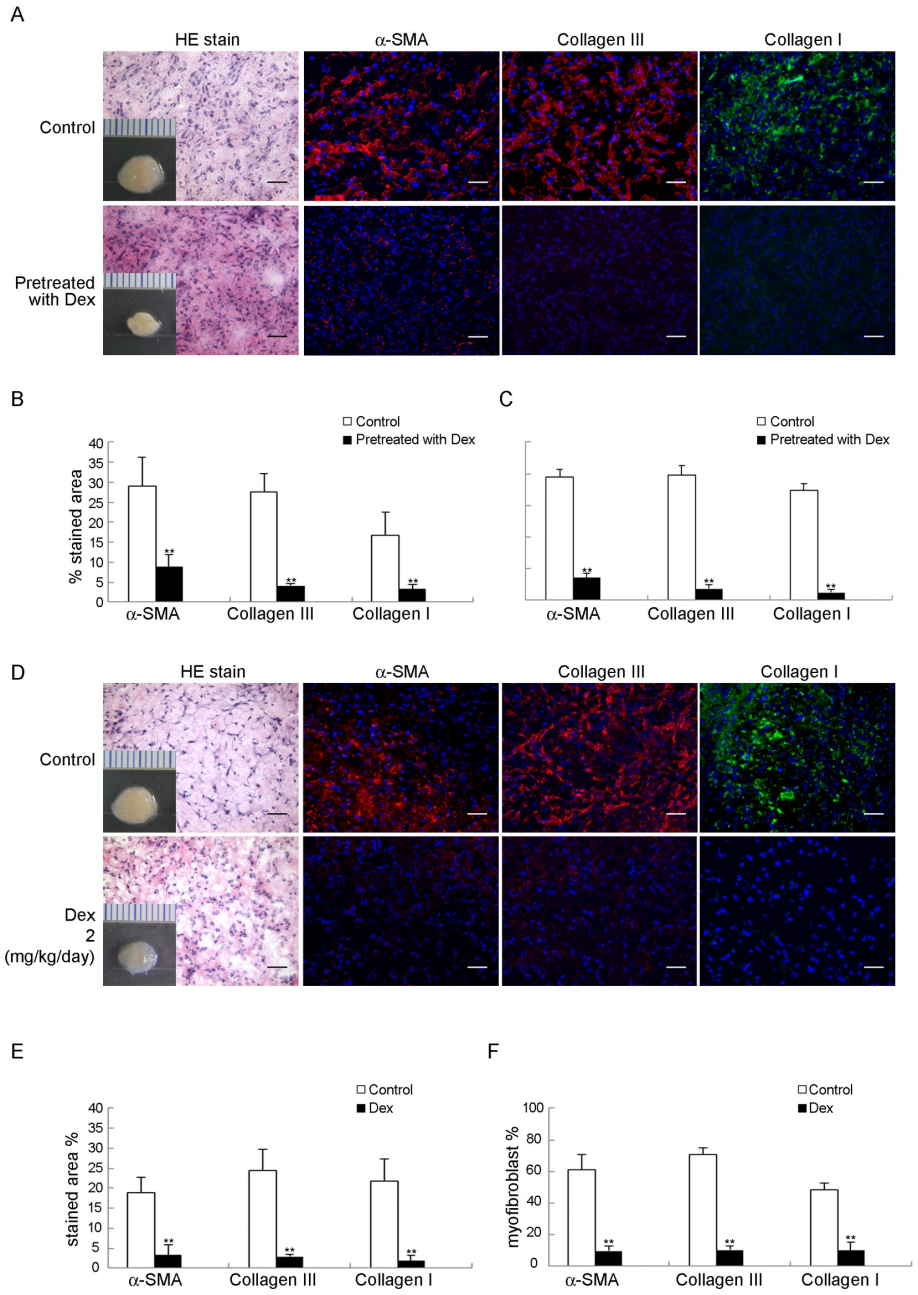
Dexamethasone inhibited FSCs formation of fibromatosis nodule in murine model. (A–C) FSCs treated with 200 nM dexamethasone (Dex) for 3 days were then delivered with Matrigel, followed by transplantation beneath the dorsal skin of nude mice. (A) Macroscopic views of the transplants after 14 days in vivo. Scale = 1 mm. H&E staining and immunofluorescence staining for α-SMA, types III and type I collagen were performed. Bars = 50 μm. (B) The percentages of stained areas. (C) The percentages of myofibroblasts. (D–F) FSCs were delivered in Matrigel and transplanted under beneath the dorsal skin of nude mice. After 7 days, dexamethasone (2 mg/kg/day) dissolved in saline was injected subcutaneously daily for 1 week, and the control group received daily subcutaneous injections of 40 ml of saline alone for 1 week (D) Macroscopic views of the implants at 14 days of implantation in vivo. Scale = 1 mm. H&E staining and immunofluorescence staining for α-SMA, type III and type I collagen. Bars = 50 μm. (E) The percentages of stained areas. (F) The percentages of myofibroblasts. Data are shown as mean ± SD (n = 3). **, *p*<0.01 denotes statistical significance. All experiments were repeated with FSCs isolated from three different donors.

To determine the effect of dexamethasone on myofibroblastic differentiation of FSCs in a murine model, FSCs were treated with dexamethasone before or after implantation with Matrigel. Treatment with dexamethasone decreased the expression of α-SMA, type III and type I collagen compared to the control group (Fig 2A–2F). All of these data suggested that dexamethasone inhibited formation of fibromatosis nodule by FSCs in vivo.

### Dexamethasone inhibits Smad family in vitro

**Fig 3:**
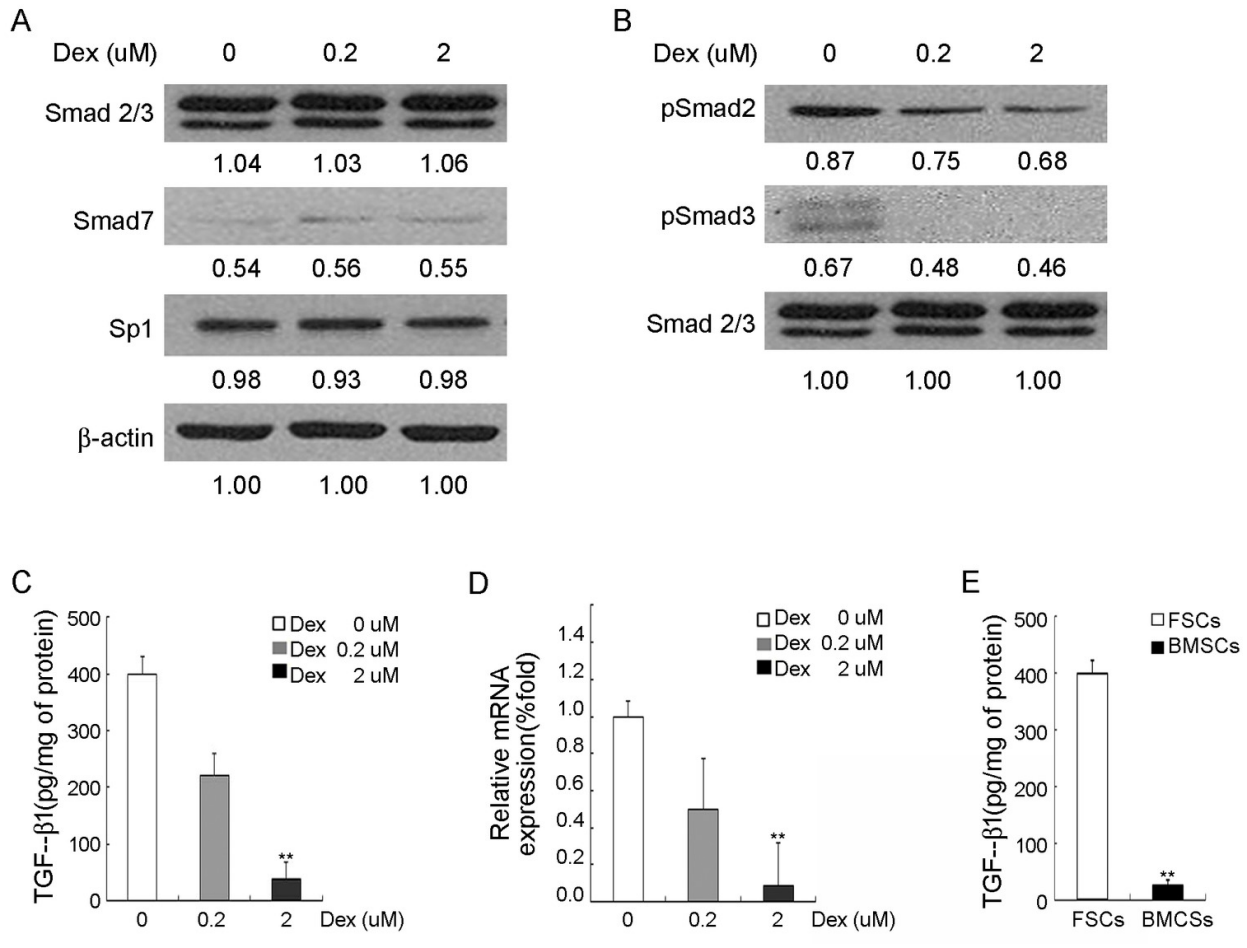
Inhibition of TGF-β1 signaling, Smad family and down-regulation of TGF-β1 in *in vitro* dexamethasone-treated FSCs. FSCs were treated with 0 μM, 0.2 μM, and 2 μM dexamethasone (Dex) for 3 days, followed by (A) and (B) Western blotting analysis of Smad family and Sp1. Immunoblotting of ß-actin & Smad2/3 was performed to show equal protein loading. (C) TGF-β1 protein levels, as measured by enzyme-linked immunosorbent assay (ELISA), in the conditioned media (D) quantitative RT-PCR analysis for mRNA expression of TGF-β1, where GAPDH was used as normalization control. (E) TGF-β1 protein levels between FSCs and BMSCs, as measured by enzyme-linked immunosorbent assay (ELISA), in the conditioned media. Data are shown as mean ± SD (n = 3). **, *p*<0.01 denotes statistical significance. All experiments were repeated with FSCs isolated from three different donors.

Since TGF-β1/Smad signaling has been shown to be involved in upregulation of α-SMA, Col3A1 and Col1A3 expression [13], we examined whether Smad signaling was affected after 3 days of dexamethasone treatment. The western blot analysis demonstrated that pSmad 2/3 level was suppressed by dexamethasone in a dose-dependent manner, while there was no obvious suppression or enhancement of Smad 7 and Sp1 by dexamethasone (Fig 3A and 3B). Dexamethasone-mediated suppression was not associated with inactivation of Sp1 or with increase in Smad 7. These data suggested that dexamethasone inhibited TGF-β1/Smad 2/3 signaling.

### Dexamethasone inhibits TGF-β1 expression of FSCs

Because Samd 2/3 can be activated by TGF-β1 [14], we examined whether TGF-β1 expression was affected by dexamethasone by enzyme-linked immunosorbent assay (ELISA) (Fig 3C) and quantitative RT-PCR (Fig 3D). We found that the TGF-β1 expression of FSCs was significantly suppressed by dexamethasone. Since TGF-β1 level in palmar fibromatosis tissue is higher than normal palmar fascia tissue [6], we compared the TGF-β1 expression by FSCs with bone marrow stem cells (BMSCs). Using enzyme-linked immunosorbent assay (ELISA), we found that the TGF-β1 expression by FSCs is higher than BMSCs (Fig 3E).

### Involvement of TGF-β1 in dexamethasone-mediated inhibition of fibromatosis

**Fig 4:**
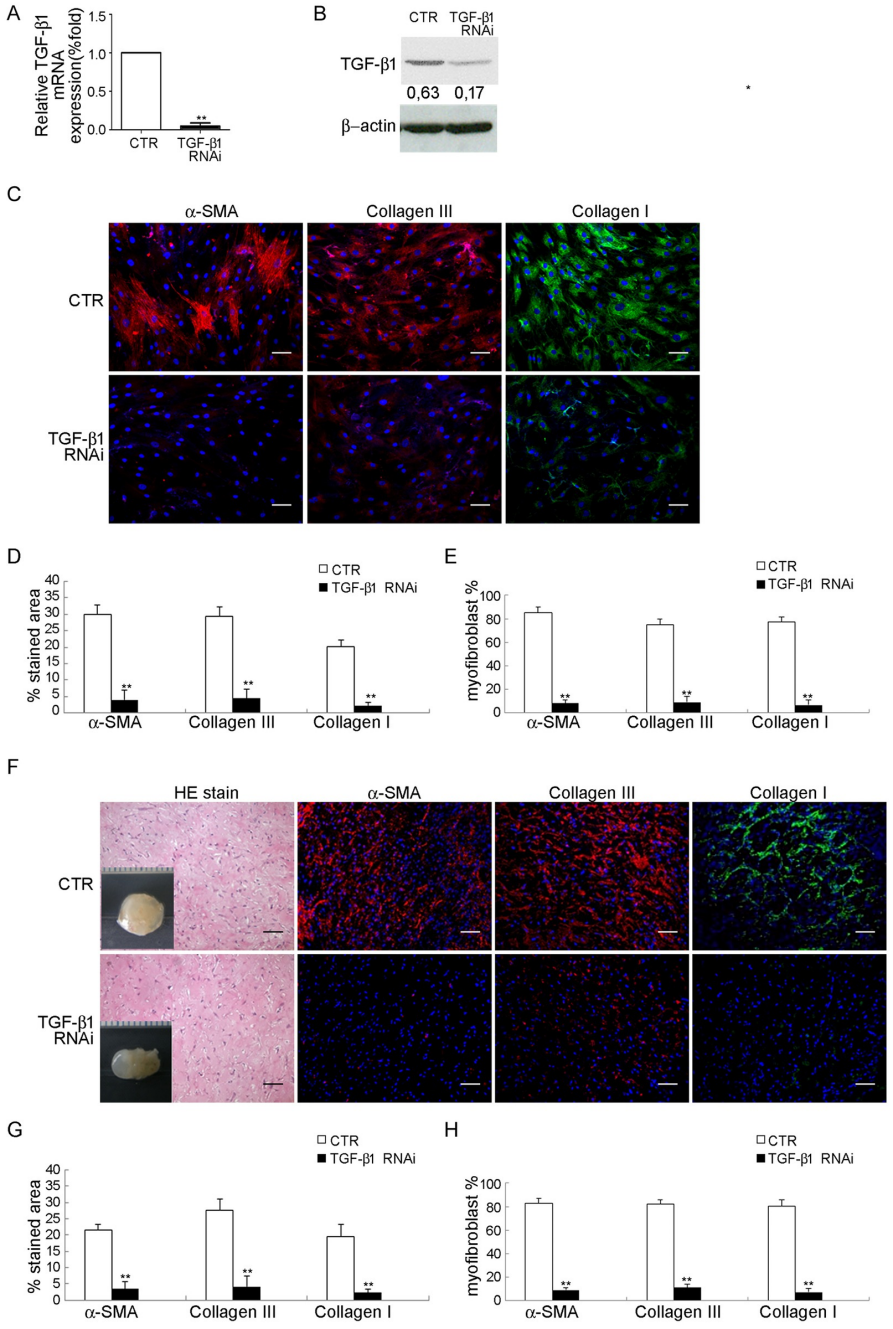
Inhibition of fibromatosis nodule formation through TGF-β1 knockdown of FSCs. Validation of knockdown efficiency by (A) quantitative RT-PCR analysis and (B) Western blotting for mRNA and protein expression of TGF-β1, respectively after transfection with a lentiviral vector carrying RNAi targeting TGF-β1 or non-targeting RNAi (CTR) for 2 days. (C–E) Transfection with a lentiviral vector carrying RNAi targeting TGF-β1 gene or non-targeting RNAi (CTR) for 14 days. (C) Immunofluorescence staining for α-SMA, type III and I collagen. FSCs were cultured for 14 days. Bars = 50 um. (D) The percentages of stained areas. (E) The percentages of myofibroblasts. (F–H) FSCs were transfected with a lentiviral vector carrying RNAi targeting TGF-β1 and non-targeting RNAi (CTR) were delivered with Matrigel and implanted beneath the dorsal skin of nude mice. Macroscopic views of the implants at 14 days of implantation in vivo. Scale = 1 mm. H&E staining and immunofluorescence staining for α-SMA, type III and type I collagen. Bars = 50 um. (G) The percentages of stained areas. (H) The percentage of myofibroblasts. Data are shown as mean ± SD (n = 3). **, p<0.01 denotes statistical significance. All experiments were repeated with FSCs isolated from three different donors.

**Fig 5:**
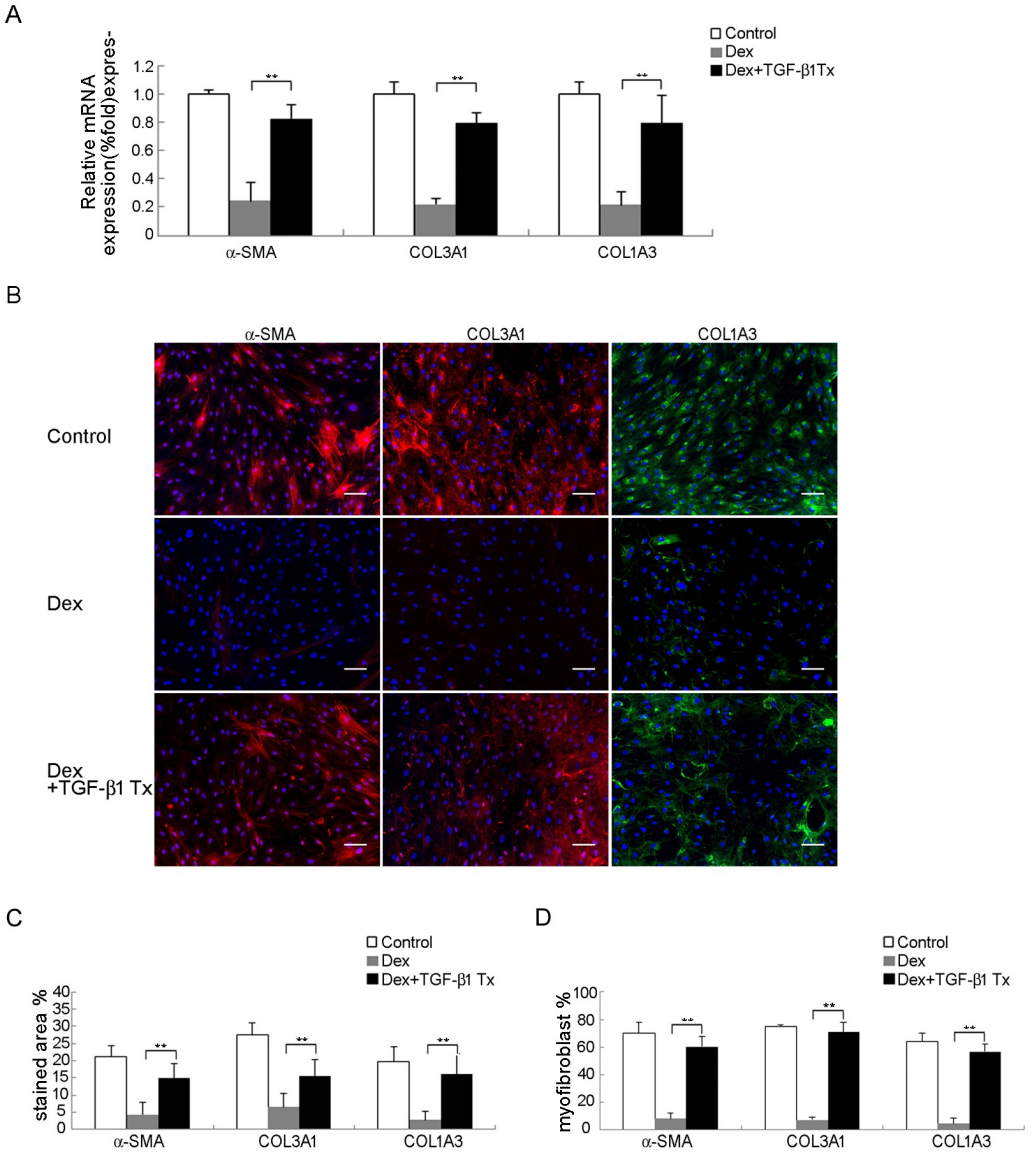
Treatment of TGF-β1 abolished TGF-β1 knockdown-mediated inhibition of myofibroblastic differentiation. (A) Quantitative RT-PCR analysis for mRNA expression of α-SMA, type III and type I collagen genes. FSCs transfected with a lentiviral vector carrying RNAi targeting TGF-β1 or non-targeting RNAi (CTR) were cultured with either 10 ng/ml TGF-β1 or saline for 14 days. (B) Immunofluorescence staining for α-SMA, type III and I collagen. FSCs transfected with a lentiviral vector carrying RNAi targeting TGF-β1 or non-targeting RNAi (CTR) were cultured with either 10 ng/ml TGF-β1 or saline for 14 days. Bars = 50 um. (C) The percentages of stained areas. (D) The percentages of myofibroblasts. Data are shown as mean ± SD (n = 3). **, *p*<0.01 denotes statistical significance. All experiments were repeated with FSCs isolated from three different donors.

To examine whether suppression of TGF-β1 expression was involved in dexamethasone-mediated inhibition of myofibroblastic differentiation in FSCs, we first blocked the function of TGF-β1 to determine if myofibroblastic differentiation of FSCs was inhibited and whether decreased pSmad2/3 is caused by decreased TGF-β1 level. FSCs were transfected with TGF-β1 RNAi and success of TGF-β1 knockdown was demonstrated using quantitative RT-PCR (Fig 4A) and western blotting analysis (Fig 4B). TGF-β1 knockdown FSCs showed decreased immunofluorescence for α-SMA and type III collagen when compared to the control both in vitro and in vivo (Fig 4A–4H). Treatment with 10 ng/ml TGF-β1 significantly attenuated immunofluorescence of α-SMA, type III and type I collagen induced by dexamethasone, when compared to the control (Fig 5). These results suggested that TGF-β1 plays a significant role during myofibroblastic differentiation of FSCs, and dexamethasone may inhibit myofibroblastic differentiation of FSCs through inhibition of TGF-β1 expression.

## Discussion

Although corticosteroids are used to treat early-stage palmar fibromatosis [11], the inhibitory mechanism of dexamethasone on palmar fibromatosis remains unclear. In our current study, we demonstrated that dexamethasone inhibited the ability of FSCs to differentiate into myofibroblasts both in vitro and in vivo. More importantly, silencing TGF-β1 inhibited FSCs differentiate into myofibroblasts both in vitro and in vivo. The results demonstrated that TGF-β1 plays a significant role in progression and recurrence of fibromatosis. TGF-β1 and its signaling pathway may be potential therapeutic targets for future drug development.

Dexamethasone has been shown to inhibit migration of tendon cells by reducing alpha-smooth muscle actin gene expression [15]; our result is consistent with this finding. In our in vitro model, we demonstrated that dexamethasone inhibited myofibroblastic differentiation of FSCs through inhibition of TGF-β1 expression and its down-stream signaling such as pSmad2 and pSmad3. We also demonstrated that dexamethasone inhibited myofibroblastic differentiation of FSCs in vivo. FSCs pretreated with dexamethasone did not undergo myofibroblastic differentiation. Moreover, inhibition of myofibroblastic differentiation decreased type I collagen accumulation. These data consistently demonstrated that dexamethasone can be a potential effective treatment for early-stage fibromatosis. However, the therapeutic effect of steroids injection has been shown to have limited durable response, with patients often ultimately develop recurrence. Therapies targeting the TGF-β1 signaling pathway may further inhibit fibromatosis progression or recurrence and provide durable treatment response.

Higher level of TGF-β1 has been found to be present in fibromatosis tissue compared to normal tissues, but the mechanism is unclear [6, 7]. Our study demonstrated that endogenous TGF-β1 is increased in FSCs. This finding may explain that higher level of TGF-β1 is present in fibromatosis tissue. The ability of myofibroblastic differentiation was reported to be stronger in FSCs than BMSCs [2]. Our current study showed higher TGF-β1 expression in FSCs than in BMSCs. We postulated that FSCs have stronger myofibroblastic differentiation than or BMSCs due to increased TGF-β1 expression. This data, combined with previous finding, further support that the precursor cells of palmar fibromatosis originated from FSCs but not from BMSCs. Because trauma or surgery can cause increased TGF-β1 [16, 17], risk of recurrence of fibromatosis following these events is high. In our previous study, among fibrosis-related growth factors, such as basic fibroblast growth factor (bFGF), epidermal growth factor (EGF) and TGF-β1, exogenous TGF-β1 significantly enhanced myofibroblastic differentiation of FSCs but not BMSCs [2]. In our current study, we postulated that progression of fibromatosis is due to endogenous TGF-β1. In the absence of exogenous TGF-β1, we demonstrated increased level of endogenous TGF-β1 in FSCs; this supports our hypothesis that the ability of spontaneous myofibroblastic differentiation in FSCs is stronger than myofibroblastic differentiation in BMSCs.

The effect of TGF-β1 can be blocked by one of mediators in the pathway. For example, Trichostatin A (TSA) inhibits TGF-β1-induced signaling by suppressing Sp1 [18] but not Smad family. All-trans retinoic acid (atRA) inhibits TGF-β1-induced signaling by increasing Smad7 expression [19]. In the present study, we found that dexamethasone decreased the expression of TGF-β1 and its down-stream pSmad2 and pSmad3. These results were consistent with findings of other studies. Bolkenius et al. demonstrated that dexamethasone impaired TGF-β/Smad signaling in hepatic stellate cells (HSC) and cirrhotic fat storing cells CFSC [20]. Another study by Meisler et al. showed that the fibrogenic effect of TGF-β was blocked by dexamethasone in rat granuloma and granulation tissue fibroblasts [21]. Because differentiated myofibroblasts is resistant to dexamethasone [22], this may explain why dexamethasone is only effective in early-stage fibromatosis.

Silencing TGF-β1 was reported to be not only a promising therapeutic approach to enhance efficacy of antitumor activity [23, 24], but also for renal fibrotic disease [25] and liver fibrosis [26]. In our study, TGF-β1 was demonstrated to play a significant role in myofibroblastic differentiation of FSCs; the ability of myofibroblastic differentiation of FSCs was diminished through silencing of TGF-β1. In our previous study, the role of TGF-β1 in fibromatosis recurrence was suggested by the response of FSCs to TGF-β1 in vitro [3]. Strategy that inhibit endogenous TGF-β1 or block the TGF-β1 signaling pathway will likely decrease progression or prevent recurrence of fibromatosis. A medication that is capable of silencing pSmad2 or pSmad3 may be a potential therapy in the future not only for superficial fibromatosis but also for deep fibromatosis groups which is more aggressive and difficult to control.

There are several limitations in this study. Although palmar fibromatosis is the most common type of fibromatosis, study of other types of fibromatosis-derived stem cells is needed to establish that TGF-β1 plays a significant role in myofibroblastic differentiation and may be a common therapeutic target for all types of fibromatosis. In addition, a murine model may not accurately simulate human fibromatosis. In our murine model, the fibromatosis nodules were created subcutaneously. However, fibromatosis in human occurs only in specific areas such as the plantar or the palmar fascia. In addition, the fibromatosis-derived stem cells used in our murine model were from Asians patients. It is known that fibromatosis is rare in Asians but more prevalent in Caucasian; potential underlying genetic differences may affect the generalized applicability of our murine model.

## Conclusions

We demonstrated that dexamethasone suppresses myofibroblastic differentiation potential of FSCs in vitro and in a murine model. We also demonstrated that that silencing of TGF-β1 expression inhibits the myofibroblastic differentiation potential of FSCs in vivo. TGF-β1 and its pathway may be an important target for future research in better understanding the biological mechanisms of fibromatosis as well as for research in developing novel target therapies.

## Supporting information

S1 Table Clone information:TGF-β1 shRNA1 (TRCN0000003317) & 2 (TRCN0000003318). (DOCX)

S1 Fig FSCs were transfected with TGF-β1 shRNA1 (TRCN0000003317) & 2 (TRCN0000003318). The success of TGF-β1 knockdown was demonstrated using quantitative RT-PCR (A) and western blotting analysis (B). TGF-β1 knockdown FSCs showed decreased immunofluorescence for α-SMA, type III collagen and type I collagen when compared to the control both in vitro (C) and in vivo (D).
(TIF)
