# Supplementary material for: Corticosteroid inhibits differentiation of palmar fibromatosis-derived stem cells (FSCs) through downregulation of transforming growth factor-β1 (TGF-β1)
Source: PLoS One. 2018 Jun 26;13(6):e0198326. doi: 10.1371/journal.pone.0198326 (PMC6019676; doi:10.1371/journal.pone.0198326)
Supplement: S1 Table — (DOCX) [file pone.0198326.s001.docx]

| **Clone ID** | **TRCN0000003317** | **TRCN0000003318** |
| --- | --- | --- |
| **Clone Name** | NM_000660.x-1167s1c1 | NM_000660.x-1606s1c1 |
| **Clone Status** | cloned | cloned |
| **Vector** | [pLKO.1](http://rnai.genmed.sinica.edu.tw/file/vector/Others/02.pLKO.1-puro_Map.pdf) | [pLKO.1](http://rnai.genmed.sinica.edu.tw/file/vector/Others/02.pLKO.1-puro_Map.pdf) |
| **NM ID** | [NM_000660](http://www.ncbi.nlm.nih.gov/nuccore/NM_000660) | [NM_000660](http://www.ncbi.nlm.nih.gov/nuccore/NM_000660) |
| **NCBI Gene ID** | [7040](http://www.ncbi.nlm.nih.gov/entrez/query.fcgi?db=gene&cmd=search&term=7040) | [7040](http://www.ncbi.nlm.nih.gov/entrez/query.fcgi?db=gene&cmd=search&term=7040) |
| **Gene Symbol** | [TGFB1](http://www.genecards.org/cgi-bin/carddisp.pl?gene=TGFB1) | [TGFB1](http://www.genecards.org/cgi-bin/carddisp.pl?gene=TGFB1) |
| **TRC Gene Functional Category** |  |  |
| **Gene Description** | transforming growth factor, beta 1 | transforming growth factor, beta 1 |
| **Target Taxonomy** | [Human](http://www.ncbi.nlm.nih.gov/entrez/query.fcgi?db=Taxonomy&cmd=search&term=9606) | [Human](http://www.ncbi.nlm.nih.gov/entrez/query.fcgi?db=Taxonomy&cmd=search&term=9606) |
| **Target Sequence** | CCCGCGTGCTAATGGTGGAAA | CCGGCCTTTCCTGCTTCTCAT |
| **Oligo Sequence** | CCGGCCCGCGTGCTAATGGTGGAAACTCGAGTTTCCACCATTAGCACGCGGGTTTTT | CCGGCCGGCCTTTCCTGCTTCTCATCTCGAGATGAGAAGCAGGAAAGGCCGGTTTTT |
| **Region** | CDS | CDS |
| **refseqSameGene** | [NM_000660.4](http://www.ncbi.nlm.nih.gov/entrez/query.fcgi?db=Nucleotide&cmd=search&term=NM_000660.4) | [NM_000660.4](http://www.ncbi.nlm.nih.gov/entrez/query.fcgi?db=Nucleotide&cmd=search&term=NM_000660.4) |
| **refseqAltGeneSameSpecies** | noAltHuman | noAltHuman |
| **refseqAltSpecies** | noMouseHit | noMouseHit |

**S1 Table.**
