# Supplementary figures and images for: Corticosteroid inhibits differentiation of palmar fibromatosis-derived stem cells (FSCs) through downregulation of transforming growth factor-β1 (TGF-β1)
Source: PLoS One. 2018 Jun 26;13(6):e0198326. doi: 10.1371/journal.pone.0198326 (PMC6019676; doi:10.1371/journal.pone.0198326)

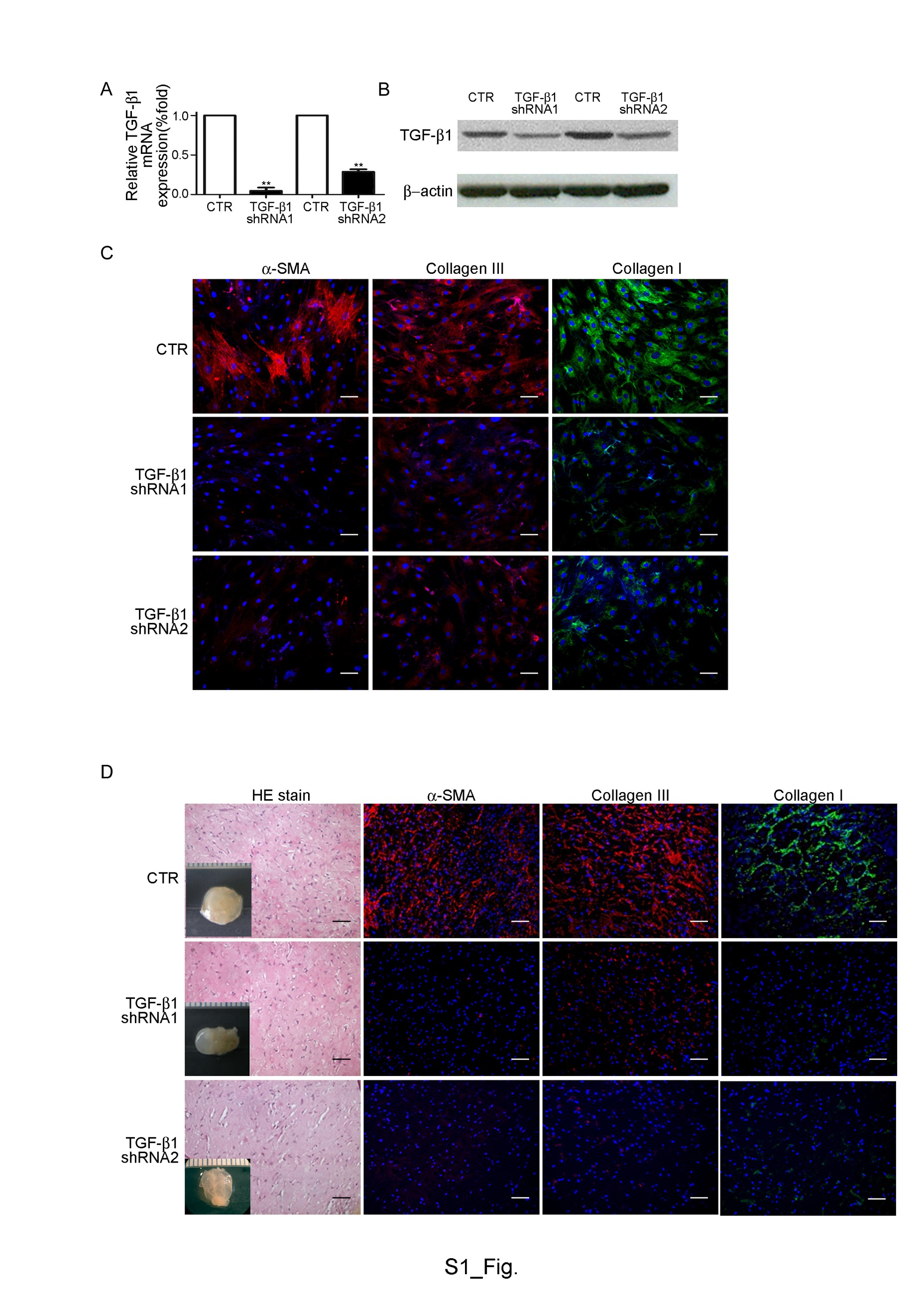

Supplement: S1 Fig — The success of TGF-β1 knockdown was demonstrated using quantitative RT-PCR (A) and western blotting analysis (B). TGF-β1 knockdown FSCs showed decreased immunofluorescence for α-SMA, type III collagen and type I collagen when compared to the control both in vitro (C) and in vivo (D). (TIF) [file pone.0198326.s002.tif]
